# A novel experimental model of erectile dysfunction in rats with heart failure using volume overload

**DOI:** 10.1371/journal.pone.0187083

**Published:** 2017-11-02

**Authors:** Fábio Henrique Silva, Frederico José Reis Veiga, Aline Gonçalves Mora, Rodrigo Sader Heck, Caroline Candida De Oliveira, Alessandra Gambero, Carla Fernanda Franco-Penteado, Edson Antunes, Jason D. Gardner, Fernanda Bruschi Marinho Priviero, Mário Angelo Claudino

**Affiliations:** 1 Hematology and Hemotherapy Center, University of Campinas, Faculty of Medical Sciences, University of Campinas, Campinas, SP, Brazil; 2 Laboratory of Multidisciplinary Research, São Francisco University Medical School, Bragança Paulista, SP, Brazil; 3 Department of Pharmacology, Faculty of Medical Sciences, University of Campinas, Campinas, SP, Brazil; 4 Department of Physiology, Louisiana State University Health Sciences Center, New Orleans, LA, United States of America; Max Delbruck Centrum fur Molekulare Medizin Berlin Buch, GERMANY

## Abstract

**Background:**

Patients with heart failure (HF) display erectile dysfunction (ED). However, the pathophysiology of ED during HF remains poorly investigated.

**Objective:**

This study aimed to characterize the aortocaval fistula (ACF) rat model associated with HF as a novel experimental model of ED. We have undertaken molecular and functional studies to evaluate the alterations of the nitric oxide (NO) pathway, autonomic nervous system and oxidative stress in the penis.

**Methods:**

Male rats were submitted to ACF for HF induction. Intracavernosal pressure in anesthetized rats was evaluated. Concentration-response curves to contractile (phenylephrine) and relaxant agents (sodium nitroprusside; SNP), as well as to electrical field stimulation (EFS), were obtained in the cavernosal smooth muscle (CSM) strips from sham and HF rats. Protein expression of endothelial NO synthase (eNOS) and neuronal NO synthase (nNOS) and phosphodiestarese-5 in CSM were evaluated, as well as NOX2 (gp91^phox^) and superoxide dismutase (SOD) mRNA expression. SOD activity and thiobarbituric acid reactive substances (TBARs) were also performed in plasma.

**Results:**

HF rats display erectile dysfunction represented by decreased ICP responses compared to sham rats. The neurogenic contractile responses elicited by EFS were greater in CSM from the HF group. Likewise, phenylephrine-induced contractions were greater in CSM from HF rats. Nitrergic response induced by EFS were decreased in the cavernosal tissue, along with lower eNOS, nNOS and phosphodiestarese-5 protein expressions. An increase of NOX2 and SOD mRNA expression in CSM and plasma TBARs of HF group were detected. Plasma SOD activity was decreased in HF rats.

**Conclusion:**

ED in HF rats is associated with decreased NO bioavailability in erectile tissue due to eNOS/nNOS dowregulation and NOX2 upregulation, as well as hypercontractility of the penis. This rat model of ACF could be a useful tool to evaluate the molecular alterations of ED associated with HF.

## Introduction

NO is well established as a mediator of penile erection. During the physiological relaxation of cavernosal smooth muscle, when NO is released from nitrergic fibers /or endothelium NO diffuses into adjacent smooth muscle cells and binds to soluble guanylyl cyclase (GCs), NO intracellular physiological receptor. Stimulation of this enzyme by NO leads to the conversion of guanosine triphosphate (GTP) in the second-messenger, cyclic guanosine monophosphate (cGMP). Elevation of cGMP activates cGMP-dependent protein kinase (PKG), which leads to reduced intracellular calcium concentration, causing smooth muscle relaxation and hence penile erection [1].

Erectile dysfunction (ED) is characterized by a persistent inability to achieve and/or maintain an erection sufficient for satisfactory sexual performance, and can affect health, relationships and overall quality of life [2]. The evident multifactorial nature of ED and the fact that ED often coexists with cardiovascular and metabolic diseases such as coronary artery disease (CAD), hypertension, obesity, dyslipidemia and diabetes mellitus has led to the conclusion that ED is predominantly a disease of vascular origin [3]. Beyond the association of ED with vascular risk factors, the ED is a stronger predictor of all-cause death and cardiovascular events including acute myocardial infarction and heart failure (HF) in patients with cardiovascular diseases [4].

HF occurs mostly as a consequence of cardiovascular diseases, leading to functional cardiac abnormalities [5]. Despite not being the main issue for treating HF patients, sexual function may lead to sexual worries which may have a negative impact on their quality of life [6]. Epidemiological studies have shown that chronic HF patients present a prevalence of ED of 61% to 89% [7,8]. Approximately 75% of male patients with HF report impaired libido [6] and 30% show complete absence of sexual activity [9]. The high prevalence of ED in HF patients have been well reported, however few have evaluated the pathophysiological alterations of the penis during HF [10]. Thus, understanding the pathophysiological alterations which lead to the development of ED associated with the HF is necessary and may assist in the development of effective ED therapies.

HF experimental models in mammals have provided additional support for understanding HF pathophysiological alterations [11]. The aortocaval fistula (ACF) model has been extensively used for studying the pathophysiology of volume-overload HF in rats [11,12]. The ACF procedure leads to an instant and constant hemodynamic change due to a reduction in arterial pressure along with a considerable enhancement in venous blood circulation to the right ventricle, resulting in an offsetting stimulation of numerous neurohormonal factors and structural modifications in the vasculature and cardiac muscle [11]. The sympathetic, angiotensin and endothelin-1 system are factors that are stimulated in response to ACF [11,13,14]. Exacerbation of these neurohormonal factors are associated with impairment of the vascular function in ACF animals and HF patients [13,15,16]. Moreover, HF-associated vascular dysregulation is thought to be due, in part, to increased oxidative stress and dysregulated NO pathways [17,18].

Alterations of the NO-cGMP signaling pathway have been implicated as a major pathomechanism for ED [1]. ED in diabetic, hypertensive, and hypercholesterolemic animals has been associated with downregulation of eNOS and nNOS expression in the penis, as well as impaired cavernosal relaxation [19–21]. Elevated oxidative stress has also been associated with ED, since superoxide anion can react with NO, reducing NO bioavailability [19,22–24]. Moreover, elevated oxidative stress and lower NO bioavailability contribute to increased contractile responses and sympathetic hyperactivity in the erectile tissue, leading to a greater difficulty in achieving penile erection [23]. As aforementioned, little is known regarding the pathophysiological alterations of the penis in HF, [10] and no detailed study has explored the role of oxidative stress and alterations in the NO signaling pathway on a model of HF-associated ED. In the present study, we hypothesized that ED in HF is associated with an imbalance between the oxidant-antioxidant system, exacerbation of the sympathetic system, and lower NO bioavailability in the erectile tissue. Our aim was to characterize the ACF rat model associated with HF as a novel experimental model of ED, as well as understand the ED pathogenesis during HF. We have undertaken functional and molecular studies to evaluate the modifications of NO signaling, sympathetic hyperactivity, and oxidative stress in the penis.

## Material and methods

### Ethics statement

All experimental procedures in this study were carried out in accordance with the general ethical guidelines for animal use established by the Brazilian Society of Laboratory Animal Science (SBCAL) and EC Directive 86/609/EEC for Animal. The experimental protocols were reviewed and approved by the Ethics Committee in Animal Research of the São Francisco University Medical School (protocol n^o^ 001.06.11). Animals were euthanized at the experimental endpoints with an overdose of sodium pentobarbital (1 mg/g) and all efforts were made to minimize animal suffering.

### Animals

All experimental procedures were carried out in accordance with the Ethical Principles in Animal Research adopted by the Brazilian College for Animal Experimentation (COBEA) and followed the Guide for the Care and Use of Laboratory Animals. This study used 40 male Sprague-Dawley rats (250–280 g) housed with free access to water and standard chow and maintained on a 12 h light–dark cycle.

### Induction of heart failure in a rat model of chronic volume overload

All the animals were anesthetized with a mixture of ketamine (50 μg/g, sc União Química Farmacêutica Nacional S/A, Embu Guaçu, SP, Brazil) and xylazine (10 μg/g, sc Hertape Calier Saúde Animal S/A, Juatuba, MG, Brazil). Heart failure was induced by surgical creation of an infrarenal aortocaval fistula (ACF) to induce chronic volume overload (VO). Briefly, a ventral laparotomy was performed to expose the abdominal aorta and vena cava and both vessels were then occluded temporarily. A short-bevel 18g needle was inserted into the abdominal aorta and advanced through the medial wall into the vena cava, creating a shunt below the renal arteries. The needle was then withdrawn and the aortic puncture site sealed with cyanoacrylate. Shunting of aortic blood into the vena cava was visually evident and indicated successful creation of VO. The musculature and skin incisions were closed by standard techniques with absorbable- and non-absorbable suture, respectively [25]. Rats were divided into two groups and subjected to VO (n = 25) or sham (n = 15) operation. Sham-operated controls received the same surgical procedure as the VO group, except that the shunt was not performed. Post-operative analgesia was provided by a single dose of flunixinme-glumine (1 mg/kg, subcutaneous) administered to the rats immediately after surgery.

### Cardiac function analysis

Cardiac ventricular dimensions and function were assessed in sedated rats (isoflurane 2%) by transthoracic echocardiography (Vevo 770; Visual Sonics, Toronto, Ontario, Canada) 1 day before the beginning of HF induction and four weeks after ACF or sham surgery. The left ventricular (LV) short-axis view was used to obtain B-mode two-dimensional images and M-mode tracings of the ventral (anterior) and dorsal (posterior) LV wall using a two-dimensional reference sector. Echocardiography provided measurements of LV internal diameter (LVID) and posterior wall thickness (LVPW) at diastole (d) and systole (s). LV systolic function was measured by fractional shortening (LVIDd − LVIDs/LVIDd) and fraction ejection. Eccentric index was evaluated by the relative wall thickness ratio 2 x LVPWd/LVIDd. All measurements were performed on three different cardiac cycles and averaged for each time point. Experimental conditions were previously described in detail [26].

### Measurement of intracavernosal pressure

Rats were anesthetized with an intraperitoneal injection of urethane (1.2 g/kg). The left carotid artery was cannulated to permit continuous monitoring of mean arterial pressure (MAP). Intracavernosal pressure (ICP) was recorded by cannulating the corpus cavernosal approximately 3–5 mm above the base of the penis, and connected to a pressure transducer. A midline abdominal incision exposed the prostate, where the right major pelvic ganglion and cavernous nerve were identified posterolateral to the prostate. The cavernous nerve was electrically stimulated with 2 platinum electrodes connected to a Grass S48 stimulator (Astro-Med Industrial Park, West Warwick, RI). Cavernous nerve stimulation was conducted at 6 V, 1 millisecond pulse width, and trains of stimuli lasting 45 seconds at varying frequencies (2–10 Hz), with intervals of 3 minutes between the stimulation trains. Changes in pressure were recorded using a PowerLab 400 data acquisition system (Chart-software, version 6.2, AD Instruments, Colorado, Springs, CO). Erectile function was calculated by the maximal ICP response during electrical stimulation (ICP at the plateau) and normalized to MAP at the time of maximum ICP (ICP/MAP), as previously described in detail [20].

### Functional studies in cavernosal strips

The penis was removed and immediately placed in a chilled Krebs solution of the following composition: NaCl, 118 mM; NaHCO_3_, 25 mM; glucose, 5.6 mM; KCl, 4.7 mM; KH_2_PO_4_, 1.2 mM; MgSO_4_ 7H_2_O, 1.17 mM and CaCl_2_ 2H_2_O, 2.5 mM. Strips of corpus cavernosum were mounted under resting tension of 5 mN in 10-ml organ chambers containing Krebs solution at 37°C (pH 7.4) and continuously bubbled with a mixture of 95% O_2_ and 5% CO_2_. Isometric force was recorded using a PowerLab 400^TM^ data acquisition system (Software Chart, version 7.0, AD Instrument, MA, USA). The tissues were allowed to equilibrate for 1 h before starting the experiments. Cumulative concentration response curves to phenylephrine (PE; 10 nM to 100 μM, α1-adrenergic receptor agonist) in basal tonus and cumulative concentration curves to sodium nitroprusside (SNP; 10 nM to 1 mM, nitric oxide donor) were obtained in cavernosal strips precontracted with PE 10 μM, as previously described in detail [20].

### Data analysis of functional assays

Nonlinear regression analysis to determine the pEC_50_ was carried out using GraphPad Prism (GraphPad Software Inc., San Diego, CA, USA) with the constraint that F = 0. All concentration–response data were fitted to a logistic function in the form: E = Emax/([1 + (10c/10x)n] + F), where E is the maximum response produced by agonists; c is the logarithm of the EC_50_, the drug concentration that produces a half-maximal response; x is the logarithm of the concentration of the drug; the exponential term, *n*, is a curve fitting parameter that defines the slope of the concentration–response line, and F is the response observed in the absence of added drug. Relaxing responses were calculated as percentages of the maximal changes from the steady-state contraction produced by phenylephrine. Data are shown as the percentage of relaxation of *n* experiments, expressed as the mean values ± SEM, as previously described in detail [27].

### Electrical-field stimulation (EFS)

Electrical field stimulation was applied in cavernosal strips placed between two platinum ring electrodes connected to a grass S88 stimulator (Astro-Med Industrial Park, RI, USA). EFS was conducted at 50 V, 1-ms pulse width and trains of stimuli lasting 10s at varying frequencies (1–32 Hz). In order to study nitrergic relaxations, cavernosal tissues were pretreated with guanethidine (30 μM) and atropine (1 μM) to deplete the catecholamine stores and to block the cholinergic receptors, respectively, as previously described in detail [22].

#### Protein extraction and Western blot analysis

For the evaluation of protein expression corpus cavernosum rat fragments were homogenized in solubilizing buffer at 4°C [1% Triton X-100, 100 mM Tris-HCl (pH 7.4), 100 mM sodium pyrophosphate, 100 mM sodium fluoride, 10 mM EDTA, 10 mM sodium orthovanadate, 2.0 mM PMSF, and 0.1 mg aprotinin/ml]. Insoluble material was removed by centrifugation at 9,000 g for 20 min at 4°C. The protein concentration of the supernatant was determined by the Biuret method. Extracts were boiled in Laemmli sample buffer containing 100 mM dithiothreitol for 5 min prior to sodium dodecyl sulfatepolyacrylamide gel electrophoresis (SDS-PAGE) using a Bio-Rad miniature slab gel apparatus (Mini-Protean). For immunoblot experiments, 25 μg of the protein extracts from each tissue were separated by SDS-PAGE, transferred to nitrocellulose membranes and incubated with antibodies anti-eNOS, anti-nNOS and anti-PDE5A (1:1000; Abcam Inc, Cambridge, MA, USA). Membranes were developed using a commercial chemiluminescence kit (secondary antibody 1:5000; GE Healthcare, UK), and signal intensity was quantified by optical densitometry (Scion Image software, ScionCorp, Frederick, MD, USA) from the developed autoradiographs. Primary antibody and secondary antibody complex were removed by incubating the blot in the striping buffer (10 Mm Tris-HCl, 100 mM β-mercaptoethanol, 8 M urea, 10% BSA) for 60 min at 60°C. After this procedure, the blots were reprobed with the antibody anti-PDE5A, anti-eNOS (1:1000; Abcam Inc, Cambridge, MA, USA) or anti-α-tubulin (1:1000; Santa Cruz Biotechnology, Santa Cruz, CA, USA).

### Polymerase chain reaction

Total ribonucleic acid (RNA) was extracted with Trizol Reagent (Gibco-BRL, Gaithersburg, MD, USA) from corpus cavernosum rat samples. RNA samples of 3 μg were incubated with 1 U deoxyribonuclease I (DNase-I) (Invitrogen, Rockville, MD) for 15 minutes at room temperature (RT) and EDTA was added to a final concentration of 2 mM to stop the reaction. The DNase-I enzyme was subsequently inactivated by incubation at 65°C for 5 minutes. DNaseI-treated RNA samples were then reverse transcribed with Superscript III and Ribonuclease (RNaseOut) (Invitrogen Corporation, Carlsbad, CA) for 50 minutes at 50°C, 15 minutes 70°C. cDNA samples were quantified using a Nanodrop spectrophotometer (ND-1000; Nanodrop Technologies, Inc, Wilmington, DE). Synthetic oligonucleotide primers were designed to amplify cDNA for the genes encoding the subunit of NADPH oxidase NOX2 (gp91^phox^), superoxide dismutase 1 (SOD-1) and GAPDH (PrimerExpressTM; Applied Biosystems, Foster City, CA, USA). The primer sequences are listed in Table 1. All samples were assayed in a 12 μL volume containing 3 μl of 10 ng cDNA, 6 μl SYBR Green Master Mix Polymerase Chain Reaction and 3 μl of specific primers in a MicroAmp Optical 96-well reaction plate using the 7200 Sequence Detection System (Applied Biosystems, Foster City, CA). The threshold cycle (Ct) was defined as the point at which the fluorescence rises appreciably above the background fluorescence. The dissociation protocol was performed at the end of each run to check for nonspecific amplification. All samples were amplified in duplicate, and the mean of the threshold cycle was used for further calculations. The probe signal was normalized to an internal reference, and GAPDH was used as a reference gene; the fold increase was calculated by the conventional comparative CT (DDCt) method, as previously described in detail [20].

**Table 1:** Sequence and primer concentration for each primer pair using quantitative RT-PCR.

Gene	Primer Sequence	Primer concentration (μM)
NOX2 (GP91^phox^)—F	5’-CATGCTGATCTTGCTGCCAGT -3’	300
NOX2 (GP91^phox^)—R	5’-TGTCTTCGAATTCTGGTTGAG -3’	300
SOD-1 –F	5’ TCAATATGGGGACAATACACAAG 3’	150
SOD-1 –R	5’ GGACCGCCATGTTTCTTAGA 3’	150
GAPDH–F	5’- CCTGCCAAGTATGATGACATCAA -3’	50
GAPDH–R	5’- AGCCCAGGATGCCCTTTAGT-3’	50

### Redox state evaluation

Redox state was assessed measuring superoxide dismutase (SOD) activity and malondialdehyde (MDA) concentration in plasma as an index of the lipid peroxidation. For SOD activity, plasma was kept on ice for assaying and samples, standards and all reagent enzymes were prepared and performed as described in the commercial kit (Superoxide Dismutase Assay Kit, Cayman Chemical Co., Ann Arbor, MI, USA). Lipid peroxidation was evidenced by the formation of MDA as the product of thiobarbituric acid reactive substances (TBARS), samples, standards and all reagent enzymes were prepared and performed as described by the manufacturer’s instructions (TBARS Assay Kit, Cayman Chemical, Ann Arbor, MI, USA). The values were expressed as mmol/L of MDA.

### Drugs

All reagents used were of analytical grade:.Atropine, guanethidine, Nω-Nitro-L-arginine methyl ester hydrochloride (L-NAME), phenylephrine, sodium nitroprusside, urethane (Sigma Chem Co., St. Louis, MO, USA), Funixinme glumine (Chemitec Agro-Vetrinária S/A, Jacarei, SP, Brazil), Isoflurane, Ketamine (União Química Farmacêutica Nacional S/A, Embu Guaçu, SP, Brazil) and xylazine (Hertape Calier Saúde Animal S/A, Juatuba, MG, Brazil).

### Statistical analysis

Data are expressed as mean ± SEM of *n* experiments. In the cumulative concentration- and frequency–response curves, data are expressed as mean of the contraction in mN divided by weight of cavernosal tissue (mN/mg of tissue) ± SEM of n experiments. The program Instat (GraphPad Software) was used for statistical analysis. Statistical comparisons carried out using Student's unpaired t-test. A value of P<0.05 was accepted as significant.

## Results

### Animal parameters

No significant differences after surgery were observed in the body weights of HF- and sham-operated rats (Table 2). Rats with HF display an increase in the total cardiac mass and isolated left ventricle mass (P<0.05) however there were no changes in mean arterial pressure (MAP) when compared to sham rats (Table 2).

**Table 2:** Body weight, mean arterial pressure, total cardiac mass, left ventricle mass from control (Sham) and heart failure (HF) groups.

	Body weight (g)		Mean arterial pressure (mmHg)	Cardiac mass total (g)	Isolated Left ventricle mass (g)
Groups	Initial	Final	Final	Final	Final
**SHAM**	301 ± 12	417 ± 15 (10)	91 ± 11 (10)	1.55 ± 0.02	1.14 ± 0.01
**HF**	308 ± 4	403 ± 14 (12)	105 ± 15 (12)	2.31 ± 0.23 *	1.65 ± 0.14*

Data represent the means ± SEM for 10–12 animals.

* P<0.05 compared to sham group

### HF produces ventricular dilation and cardiac dysfunction

**Fig 1:**
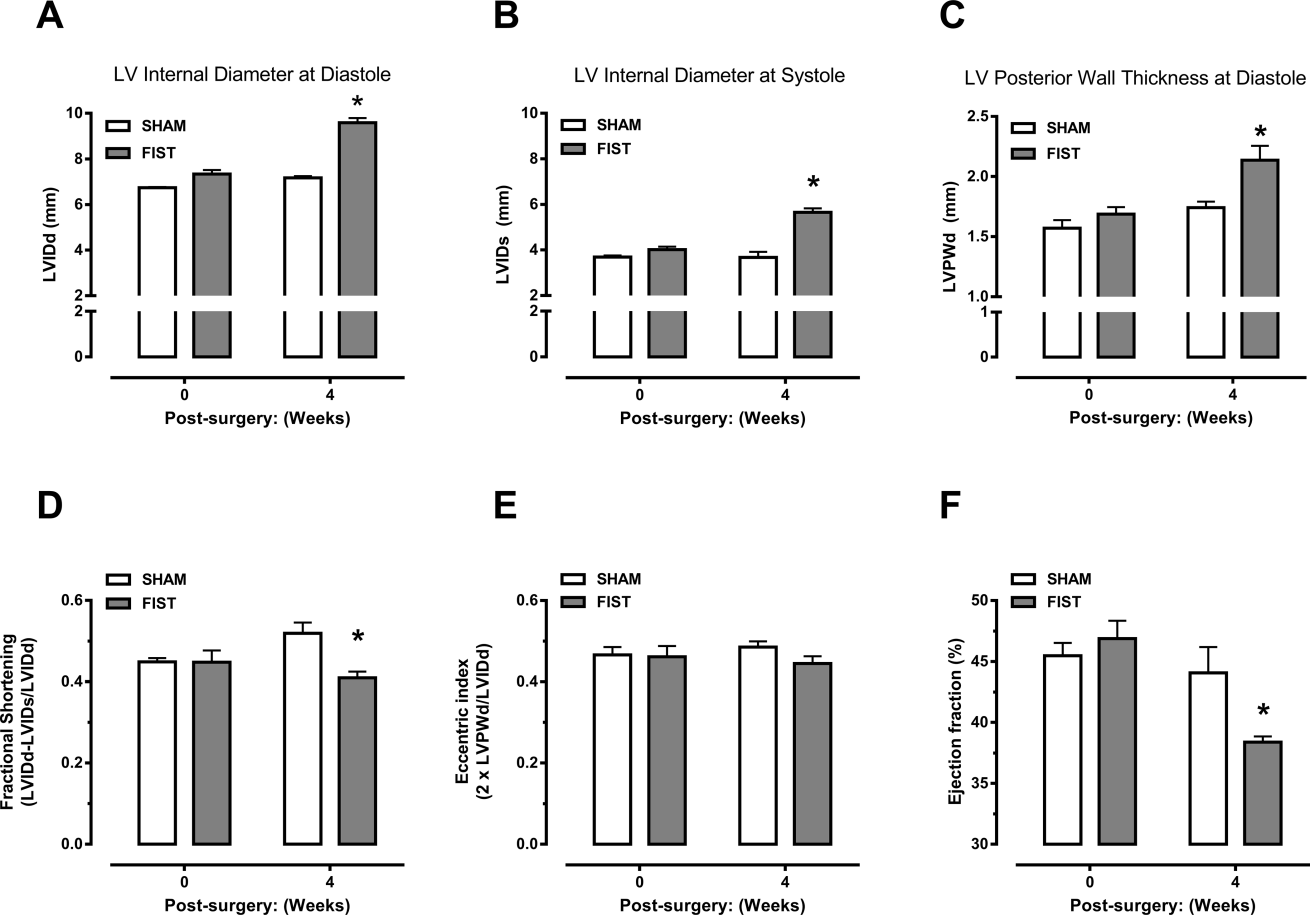
Echocardiographic exploration of cardiac function in sham (n  =  10) and heart failure (HF; n  =  10) rats. After 4 weeks, heart failure rats presented increased left ventricle (LV) internal diameters at diastole (panel A), LV internal diameters at systole (panel B) and LV posterior wall thickness at diastole (panel C) and reduced fractional shotering (panel D) and fractional ejection (panel F). Data are mean ± SEM of n experiments. *P<0.05 compared to sham group.

Echocardiographic assessment of LV structure demonstrated that rats with HF developed a progressive increase in LVIDd and LVIDs with significant dilation evident to the 4 weeks endpoint (34% and 53% respectively; Fig 1A and 1B). These rats also exhibited an increase in LVPWd with significant wall thickening occurring by 4 weeks (23%; Fig 1C). The eccentric index was not changed in HF rats (Fig 1E). However, rats with HF displayed decreased ejection fraction and decreased shortening fraction values compared to the sham group (Fig 1D and 1F), characterizing a cardiac dysfunction in this animal model. Thus, at the endpoint, HF was confirmed in approximately 75% of the animals submitted to VO, and the failure rate in HF induction process was approximately 25% where the rats died or did not develop HF.

### HF rats display erectile dysfunction

**Fig 2:**
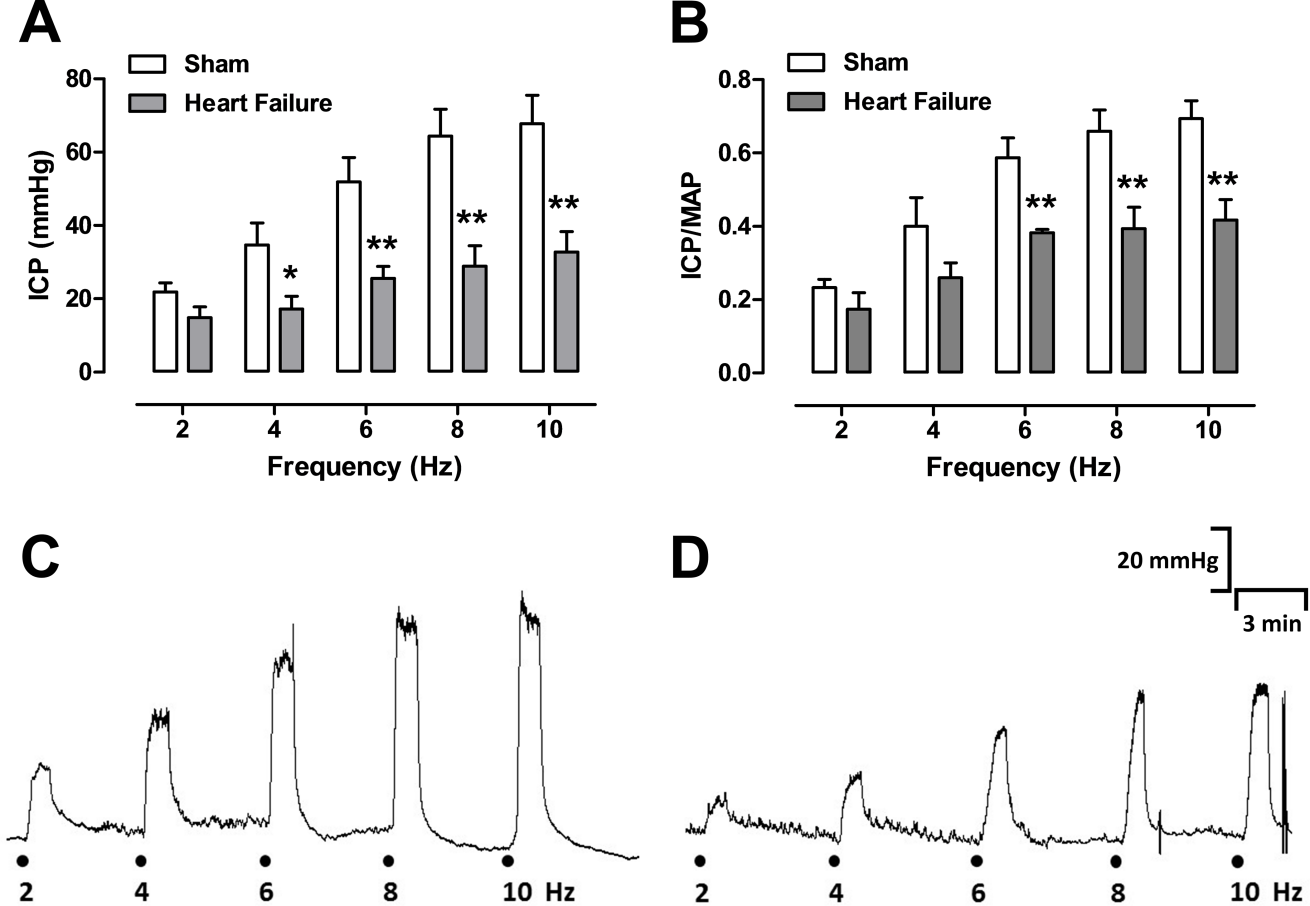
Measurements of rat intracavernous pressure (ICP) obtained by stimulation of the cavernous nerve at 2–10 Hz. Data were obtained from the sham group and the heart failure group, and are shown either as peak ICP (mmHg; panel A) or ICP divided by mean arterial blood pressure (ICP/MAP ratio; panel B). Representative trace of ICP from sham (panel C, n = 5) and heart failure (panel D, n = 5) groups. Data are mean ± SEM of n experiments. **P<0.01 and *P<0.05 compared to sham group.

No significant changes were observed in the basal ICP (mmHg) or basal ICP/MAP ratio in the HF- and sham rats. Cavernous nervous stimulation (2–10 Hz) caused a frequency-dependent increase in either the ICP peak (mmHg) or the ICP/MAP ratio in all groups. ICP peaks in HF rats were significantly lower (P<0.05) compared with sham rats, particularly at 4, 6, 8 and 10 Hz (Fig 2A). When ICP was normalized to MAP, similar data were obtained in HF rats (Fig 2B) in the frequencies of 6, 8 and 10 Hz, suggesting that HF leads to ED *in vivo*. Typical traces of ICP in sham and HF rats are shown in Fig 2C and 2D, respectively).

### HF rats display nitrergic cavernosal relaxations dysfunction

**Fig 3:**
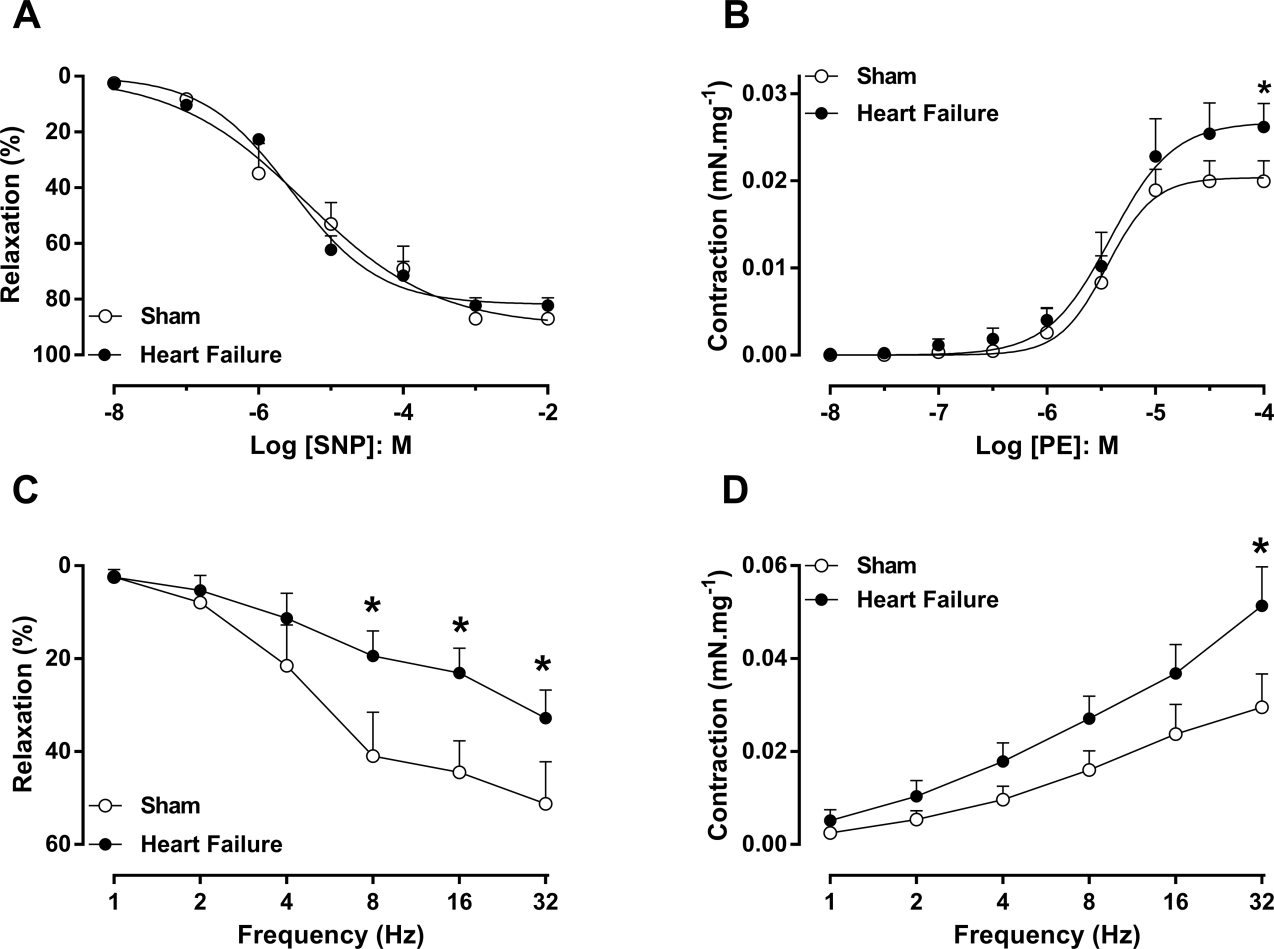
Concentration response curves to SNP (panel A) and frequency response curve to electrical field stimulation (nitrergic response; 1 = 32Hz; panel C) in corpus cavernosum strips from sham (n = 5 and 6, respect.) and heart failure (HF; n = 6 and 5, respect.) rats, precontracted with phenylephrine (PE). Concentration response curves to PE (panel B), frequency response curve to electrical field stimulation (neurogenic response; 1-32Hz; panel D) from sham (n = 7 and n = 7, respect.) and HF (n = 7 and n = 6, respect) rats in basal tonus. Experimental values for SNP and nitrergic relaxation were calculated relative to the maximal changes from the precontraction produced by PE in each tissue, which was considered 100%. Experimental values to PE and neurogenic response were calculated in mN/mg. Data represent the means ± SEM of n experiments. *P<0.05 compared with the sham group.

EFS of cavernosal tissues pretreated with guanethidine (3x10^-5^ M) and atropine (10^−6^ M) caused frequency-dependent rat corpus cavernosum relaxations in both groups (Fig 3B). A marked decrease in the relaxations of cavernosal tissues of HF rats in comparison with the sham group was observed, particularly at 8, 16 and 32 Hz (Fig 3C).

### Concentration–response curve to SNP in cavernosal tissue

The cumulative addition of NO donor, SNP (10^−8^ to 10^−2^ M) to PE-contracted (10^−5^ M) tissues produced concentration-dependent cavernosal relaxation, however no significant differences were observed between sham and HF groups (Fig 3A; Table 3). The potency (pEC_50_) and maximal responses (E_max_) values are represented in Table 3.

**Table 3:** Potency (pEC_50_) and maximal responses (E_max_) values obtained from concentration-response curves to sodium nitroprusside (SNP) and phenylephrine in corpus cavernosum from control (Sham) and heart failure (HF) groups.

	Phenylephrine		SNP	
Groups	pEC_50_	E_max_ (mN/mg)	pEC_50_	E_max_ (%)
**Sham**	5.45 ± 0.04	0.019 ± 0.001	5.35 ± 0.14	87 ± 4
**Heart failure**	5.41 ± 0.05	0.027 ± 0.001*	5.55 ± 0.09	82 ± 3

Potency is represented as -log of the molar concentration to produce 50% of the maximal relaxation response elicited by the agonist relative contractile response produced by phenylephrine (10 μM), which was considered 100%. Data represent the mean ± S.E.M. of 5–7 experiments.

*P<0.05 compared to sham group.

### HF rats display sympathetic hyperactivity and hypercontractility in erectile tissue

The α1-adrenoceptor agonist phenylephrine (10^−8^ to 10^−4^ M) induced concentration-dependent corpus cavernosum contractions in both sham and HF rats (Fig 3B). The maximal responses (Emax) were significantly greater (P <0.05) in the corpus cavernosum of HF, in comparison with sham rats (Fig 3B, Table 3). No significant differences in pEC50 for phenylephrine were found between groups (Fig 3C, Table 3).

EFS produced frequency-dependent contractions in cavernosal strips (2–32 Hz) in all groups (Fig 3D). EFS-induced contractions were fully abolished by sympathetic inhibitory drug guanethidine (3 × 10^−5^ M), confirming that nerve-induced cavernosal contractile responses are mediated by noradrenaline release. In the cavernosal strips of HF rats, EFS-induced contraction was enhanced by 70% at 32 Hz, in comparison with that observed in sham rats (Fig 3D).

### Reduction of the protein expression for eNOS, nNOS and PDE5 in the corpus cavernosum

**Fig 4:**
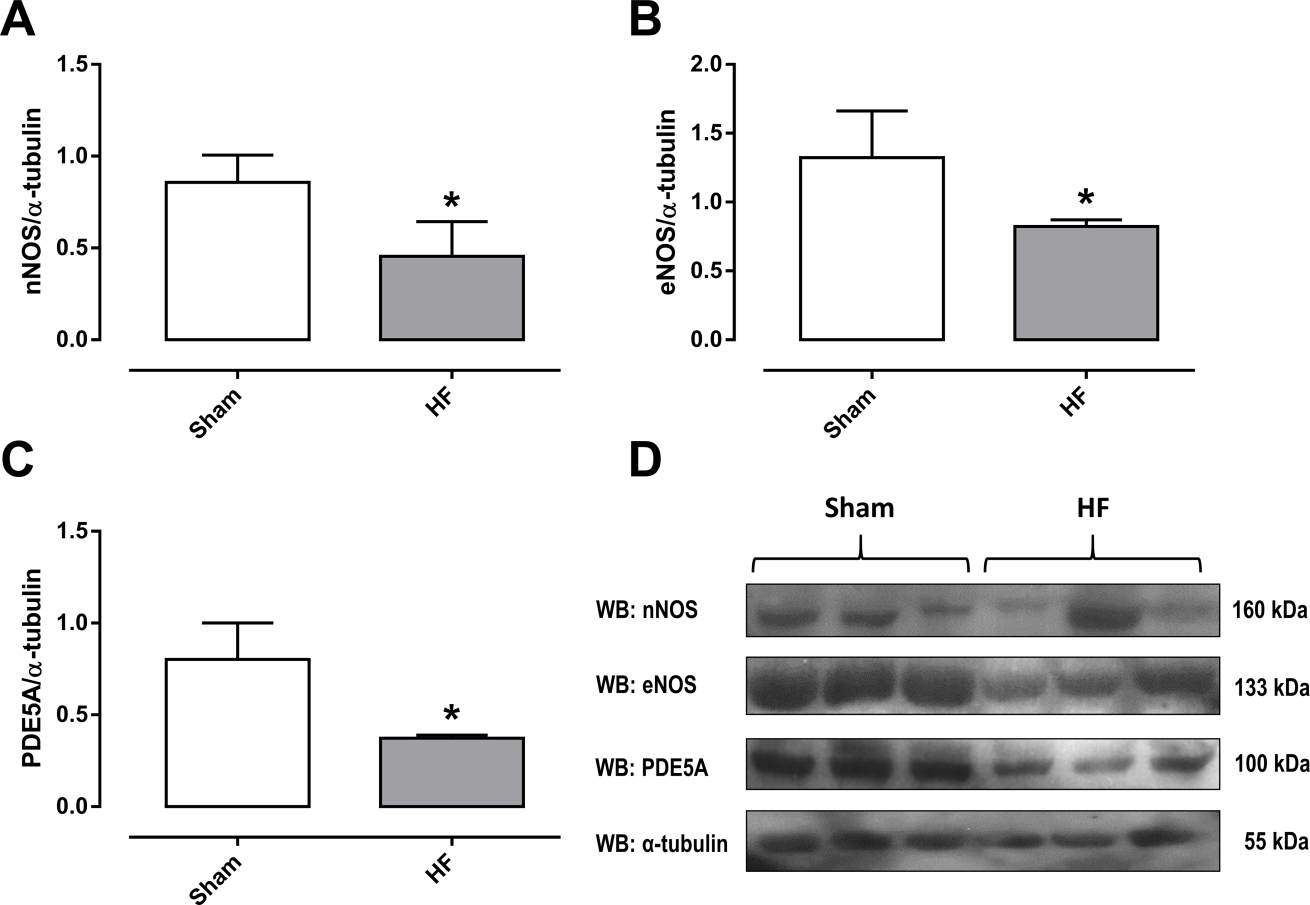
Total protein expressions of nNOS (panel A), eNOS (panel B) and PDE5A (panel C) in the corpus cavernosum of sham and heart failure (HF) rats. Representative densitometric of western blotting for the nNOS, eNOS and PDE5A in homogenates of corpus cavernosum from rats sham or HF (Panel D), which were normalized by α-tubulin levels. WB: western blotting. Data represent the means ± SEM of 3 experiments in each group. *P<0.05 compared with the sham group.

Considering that NO-cGMP pathway is established as a mediator of penile erection, and HF rats display erectile dysfunction and nitrergic cavernosal responses, we evaluated the protein expression of eNOS, nNOS and PDE5. The protein expression for eNOS, nNOS and PDE5 enzymes was significantly reduced (P<0.05) by approximately 38%, 44% and 53%, in cavernosal tissues from the HF group in comparison with the sham group, respectively (Fig 4), suggesting an impairment in the bioavailability of NO.

### Gene expression of NOX2 and SOD in the corpus cavernosum and lipid peroxidation (T-BARs) and SOD activity in plasma

**Fig 5:**
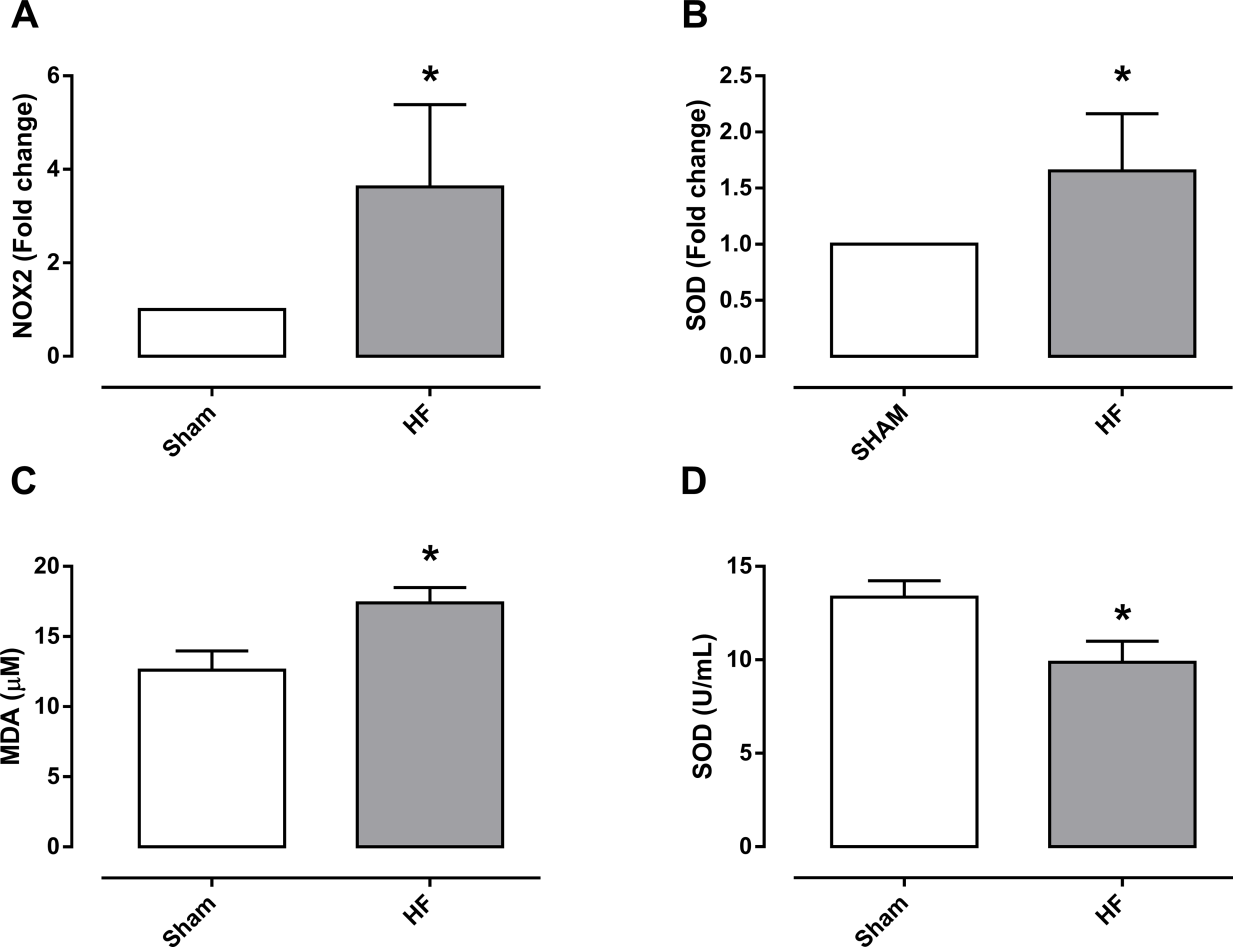
mRNA expression of NOX2 (gp91^phox^; panel A) and superoxide dismutase (SOD; panel B) in the homogenates of the corpus cavernosum from sham and heart failure (HF) rats. The mRNA expression level of each gene was normalized by GAPDH expression. Evaluation of oxidative damage through the quantification of malondialdehyde (MDA, panel C) and plasma enzyme SOD activity (panel D) of sham and heart failure (HF) rats. Data represent the means ± SEM of 4–6 experiments in each group. *P<0.05 compared with the sham group.

The mRNA expression for the NOX2 and SOD in cavernosal tissues was approximately 3.6- and 1.7-fold higher (P<0.05) in the corpus cavernosum of HF group, compared with the sham group, respectively (Fig 5A and 5B).

In plasma from HF group, MDA levels (lipid peroxidation) were significantly higher (P<0.05) compared with the sham group (Fig 5C). In accordance with this finding, plasma SOD activity was significantly lower in the HF group compared with the sham group, suggesting an imbalance in the redox system (Fig 5D).

## Discussion

HF is a disorder that has many different causes and involves diverse physiological systems [5]. The ACF model has been used to better understand the complex HF pathophysiology [11–14,25]. Our data show that, after 4 weeks, the rats submitted to ACF showed an increase in the total cardiac mass and isolated left ventricle mass, suggesting that these animals presented cardiac hypertrophy due to volume overload. In HF, reduction of the amount of myocyte and augmented myocardial tension lead to hypertrophy of the remaining myocytes, both directly and through an increase in the levels of neurohormonal factors, leading to left ventricular remodeling and fibrosis [5]. Due to the cardiac hypertrophic process in our animals, we decided to perform a transthoracic echocardiographic analysis to evaluate the LV diastolic and systolic function. Echocardiographic assessment of the LV structure demonstrated that rats submitted to ACF developed a progressive increase in LVIDd and LVIDs followed by increased wall thickening and significant dilation. Moreover, these rats showed a reduction in the shortening fraction and ejection fraction, demonstrating that the rats used in our study had developed HF. Lower ejection fraction by the ventricle is an important feature of HF [28]. Previous studies have shown similar cardiovascular changes using the ACF model in rats after 4, 8 and 12 weeks [12,26].

HF is associated with endothelial dysfunction, characterized by impaired endothelium-dependent relaxation in the blood vessels of both human [29] and animal HF models [11,13,15]. Some authors have proposed concepts such as ED equal to endothelial dysfunction, as a plausible explanation for the risk relationship, suggesting that this mechanism is a universal vascular derangement at the vascular penile level [30]. In our study, we evaluated the effect of HF on the erectile function of rats submitted to ACF. We initially evaluated the ICP in anesthetized rats as a direct index of penile erection. Cavernous nerve stimulation in sham rats caused frequency-dependent increases in ICP and ICP/MAP ratio, which was largely reduced in the HF group. ICP reduction suggested a dysfunction in the cavernosal vascular and/or trabecular smooth muscle leading to lower blood circulation into the erectile tissue, resulting in the development of ED. A recent study reported that rats with heart failure secondary to myocardial infarction displayed ED [10].To our knowledge, our study is the first study showing that volume overload HF rats display ED. Once ED was established in the ACF rat model, we studied the role of HF in the alterations of signaling pathways that regulate the cavernosal smooth muscle tone during the erection process.

Penile erection is a complex neurovascular process [1], which involves penile nerves, blood vessel endothelium and smooth muscle cells. The contractile state of the erectile tissue is well known to control erectile penile [31]. The balance between flaccid penis and penile erection is controlled by contractile and relaxant transmitters. Noradrenaline derived from sympathetic nerve stimulation is responsible for maintaining the penis in the flaccid state via activation of α1-adrenoceptors, and thus leading to cavernosal smooth muscle contraction [31]. To further elucidate the contractile mechanism in the corpus cavernosum of HF rats, we evaluated the response to α1-adrenoceptor agonist phenylephrine and noradrenergic contractions evoked by EFS. In the set of experiments to evaluate the noradrenergic contractions, corpus cavernosum were incubated simultaneously with the NOS inhibitor L-NAME plus nonselective muscarinic antagonist atropine to allow EFS-induced contractions caused by stimulation of adrenergic nerves fibres only. We showed that the sympathetic cavernosal contractions were significantly greater in HF rats compared to the sham group, suggesting a state of sympathetic hyperactivity in the erectile tissue. Consistent with our findings, HF patients and ACF rats displayed sympathetic hyperactivity [11,32]. Decreased nNOS expression is associated with sympathetic hyperactivity in the mesenteric artery of hypertensive animals [33]. Furthermore, we have also observed decreased NOS expression in erectile tissue from HF rats. Additionally, the maximal contractile response to the direct α1-adrenoceptor activation with phenylephrine was greater in the corpus cavernosum from the HF group. Collectively, our data suggest that sympathetic hyperactivity and hypercontractility in the corpus cavernosum of the HF group may render penile erection more difficult to achieve. A recent study showed that HF rats display increased contractile response elicited by α1-adrenergic receptor associated with upregulation of ROCK 2 and MYPT-1 phosphorylation in the corpus cavernosum, as well as sympathetic hyperactivity [10].

Considering that NO is essential for penile erection [1], we tested the hypothesis that the impairment of erectile function may also be associated with the reduction of NO bioavailability in the erectile tissue from HF rats. Penile erection is initiated by NO produced by nNOS and erection is maintained by NO produced by eNOS and nNOS [34]. In our study, the protein expression for eNOS and nNOS was significantly reduced in the corpus cavernosum from HF rats. These reductions in NOS expression are consistent with our functional studies, where nitrergic relaxation responses and ICP were lower in the corpus cavernosum of HF rats. Decreased nNOS expression has been related in hypothalamus and paraventricular nucleus of rats with HF [35,36], as well as in models of ED [21,37]. In ED animal models, decreased eNOS expression protein/mRNA is a known alteration of the corpus cavernosum [37–39], as well as in the ventricule and atrium from of HF models [40,41]. NO can be exogenously distributed to tissues and cells by various NO-generating agents [42]. We have investigated the corpus cavernosum relaxant responses to the inorganic compound NO donor SNP. In our study, SNP produced corpus cavernosum relaxation of rats in a similar manner to sham and HF groups. Therefore, injury of erectile tissue relaxant responses in HF group seems to be due to lower NO synthesis and/or NO bioavailability at the level of endothelial cells and nitrergic nerves in the penis. During the process of penile erection, the action of PDE5 is to hydrolyze cGMP to 5'GMP, thus ceasing the NO-cGMP pathway and the erectile response [31]. In the corpus cavernosum, PDE5 gene expression is positively regulated by cGMP [43]. Our results showed reduced PDE5 protein expression in the corpus cavernosum of rats with HF, suggesting a possible PDE5 downregulation due to decreased NO bioavailability. In fact, eNOS knockout mice and combined eNOS and nNOS double knockout mice exhibit lower PDE5 expression in the penis [44,45].

Oxidative stress has been reported to play an important role in the pathophysiology of HF and in independent risk factors ED- related [18,21,24,46]. The sustained increase of oxidative stress in chronic degenerative diseases is related to changes in NO bioavailability. Among the systems that participate in the formation of oxidative stress, NADPH oxidase complexes are the major formers of the superoxide anion in pathologic conditions [47]. NOX2 (gp91^phox^) is the main catalytic subunit responsible for the activation of the NADPH complex in the generation of superoxide anion [47]. Several studies have shown that gp91^phox^ is increased in experimental models of HF [48], HF patients [49], model of ED [20,24] and ED patients [50]. Our data show that rats with HF present an increase in NOX2 gene expression in the corpus cavernosum, suggesting that the NADPH oxidase contributes to ED by generating superoxide anion that reacts with NO, decreasing NO bioavailability.

SOD is known to form part of the antioxidant system and to play an important role in the control of superoxide anion excess, in particular in the vascular system, conserving the corpus cavernosum smooth muscle and endothelium functions, and promoting improvements in NO bioavailability [21,23,47]. In our study, our findings show a significant increase in the SOD gene expression in the corpus cavernosum of animals with HF. This increase in SOD gene expression may be a compensatory mechanism, due to augmented oxidative stress formation in the penis of HF rats. In fact, increased SOD expression have been observed in the corpus cavernous of ED condition and increased oxidative stress [51]. In the plasma from HF rats, we found a decrease in SOD enzymatic activity in the plasma, indicating an impairment of antioxidant system. A previous study reported that the deficiency of SOD activity results in an impairment of endothelial vascular system through inactivation of NO [52]. In fact, plasma SOD activity is reduced in patients with HF, contributing to endothelial dysfunction in these patients [53,54]. All cellular components are compromised by the action of reactive oxygen species, however, membrane components are the most affected, as a result of lipid peroxidation, leading to changes in structure and permeability [55]. Lipid peroxidation products such as MDA can be used as indicators of the action of reactive oxygen species in the organism [55]; elevated plasma levels of MDA have been associated to HF patients, [56]. In our study, animals with HF also displayed elevated plasma levels of MDA. Collectively, these data indicate that increased oxidative stress contribute to ED in rats with HF.

## Conclusion

In summary, our study shows that ACF rat model displays ED associated with imbalance between the oxidant-antioxidant system, dysregulation of NO signaling and hypercontractility of the penis. Therefore, our findings show that the ACF model is a novel experimental model for evaluating ED in HF.
